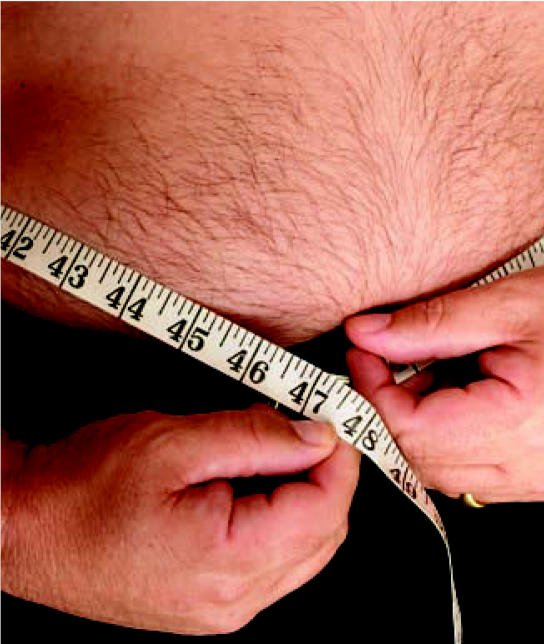# Phthalates and Metabolism: Exposure Correlates with Obesity and Diabetes in Men

**Published:** 2007-06

**Authors:** Melissa Lee Phillips

The prevalence of obesity, insulin resistance, and diabetes has increased considerably in the past few decades. Many plausible contributing factors have been identified for this increase, among them low testosterone levels in men. Research has found that exposure to certain synthetic chemicals adversely affects testicular function in animals and possibly in humans. A new analysis looked for—and found—that exposure to one class of these chemicals, phthalates, correlated with two metabolic abnormalities in men: abdominal obesity and insulin resistance **[*EHP* 115:876–882; Stahlhut et al.]**.

Phthalates are commonly used in products such as cosmetics, soaps, pesticides, lubricants, plastics, and paints. They are widespread; indeed, more than 75% of the U.S. population carries detectable levels of several phthalate metabolites. Studies have also found associations between some phthalate metabolites and antiandrogenic effects in humans, including both infant and adult males.

The authors used 1999–2002 data from the CDC National Health and Nutrition Examination Survey (NHANES) to look for a connection between phthalate exposure and metabolic disease in adult men. They compared urine concentrations of six phthalate metabolites to the participants’ waist circumference and measures of insulin resistance. The analysis controlled for a variety of potential confounders, including age, ethnicity, fat and calorie consumption, physical activity, and smoking status.

Four phthalate metabolites were significantly associated with greater waist circumference and three with increased insulin resistance. When the authors further controlled their models for measures of participants’ kidney and liver function, the associations decreased somewhat but remained significant for all but one metabolite.

The authors caution that this first look at phthalates, obesity, and insulin resistance is limited by the study’s cross-sectional design and the single measurement of urine phthalate metabolites (an imperfect measure of long-term exposure). In addition, although the study was based on the hypothesis that phthalates cause metabolic abnormalities by decreasing androgen levels or function, the authors couldn’t examine this mechanism, because the NHANES data do not contain measures of sex hormones in men. They note that other mechanisms could also be involved in a relationship between phthalates and metabolic disease.

If phthalates are eventually shown conclusively to contribute to obesity or diabetes in men, it’s still not clear how these chemicals would affect the opposite sex, since low testosterone has been associated with a lower (not higher) prevalence of metabolic disease in women. If further longitudinal studies confirm that phthalate exposure contributes to obesity, diabetes, and related disorders, actions to reduce phthalate exposure could effectively lessen the chemicals’ contribution to metabolic disorders, because phthalates are quickly metabolized and excreted by the body.

## Figures and Tables

**Figure:**